# Fluctuating seawater pH/*p*CO_2_ regimes are more energetically expensive than static pH/*p*CO_2_ levels in the mussel *Mytilus edulis*

**DOI:** 10.1098/rspb.2017.1642

**Published:** 2017-10-18

**Authors:** Stephanie Mangan, Mauricio A. Urbina, Helen S. Findlay, Rod W. Wilson, Ceri Lewis

**Affiliations:** 1 Department of Biosciences, University of Exeter, Geoffrey Pope Building, Exeter EX4 4QD, UK; 2 Plymouth Marine Laboratory, Prospect Place, West Hoe, Plymouth PL1 3DH, UK; 3 Departamento de Zoología, Facultad de Ciencias Naturales y Oceanográficas, Universidad de Concepción, Casilla 160-C, Concepción, Chile

**Keywords:** ocean acidification, natural variability, acid–base balance, metabolism, oxidative stress

## Abstract

Ocean acidification (OA) studies typically use stable open-ocean pH or CO_2_ values. However, species living within dynamic coastal environments can naturally experience wide fluctuations in abiotic factors, suggesting their responses to stable pH conditions may not be reflective of either present or near-future conditions. Here we investigate the physiological responses of the mussel *Mytilus edulis* to variable seawater pH conditions over short- (6 h) and medium-term (2 weeks) exposures under both current and near-future OA scenarios. Mussel haemolymph pH closely mirrored that of seawater pH over short-term changes of 1 pH unit with acidosis or recovery accordingly, highlighting a limited capacity for acid–base regulation. After 2 weeks, mussels under variable pH conditions had significantly higher metabolic rates, antioxidant enzyme activities and lipid peroxidation than those exposed to static pH under both current and near-future OA scenarios. Static near-future pH conditions induced significant acid–base disturbances and lipid peroxidation compared with the static present-day conditions but did not affect the metabolic rate. These results clearly demonstrate that living in naturally variable environments is energetically more expensive than living in static seawater conditions, which has consequences for how we extrapolate future OA responses in coastal species.

## 1. Introduction

Atmospheric carbon dioxide (CO_2_) levels reached 400 ppm in 2016, a rise of more than 100 ppm since the industrial revolution, and continues to rise at a current rate of 2.73 ppm yr^−1^. Projections from the representative concentration pathways (RCP) 8.5 business as usual scenario predict atmospheric CO_2_ levels will exceed 1000 ppm early into the next century [1]. The accompanying absorption of atmospheric CO_2_ by the oceans has led to a 30% increase in average global ocean pH (reduction of pH by 0.1 units) and an alteration in carbonate chemistry [2], termed ocean acidification (OA). There is now substantial evidence that OA can negatively impact the health and physiology of a wide range of marine invertebrates, with experimental OA negatively affecting over 50% of all molluscs tested to date [3,4].

While calcifying species are generally considered to be more susceptible to OA owing to the impact on calcium carbonate formation and dissolution [4,5], focus has now shifted to the impacts of OA on other physiological processes, in particular acid–base physiology and the energetic cost of ion regulation. Marine animals acutely exposed to elevated *p*CO_2_ can experience an extracellular acidosis [6]. The ability to compensate extracellular pH changes and maintain cellular homeostasis is thought to play a key role in the future survival and distribution of a species [6], and hence is considered to be a key determinant of an organism's susceptibility to future OA [7]. Marine mussels, such as *Mytilus edulis*, provide a range of ecosystem services such as habitat structure and water purification, as well as contributing an estimated $19 billion to the global economy in 2014. Previous studies in *M. edulis* revealed that hypercapnia induced extracellular acidosis [8–10] with no compensatory increase in 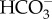, suggesting a poor ability to acid–base regulate. If uncompensated, reductions in extracellular pH have the potential to influence protein stability and enzyme function [11], leading to concerns over the future impacts of OA on this commercially important species.

The majority of experimental OA studies to date for *M. edulis* and other coastal species measure responses at the end of medium-term exposures to static seawater pH levels, using predictions based on global open-ocean averages, where pH and other carbonate chemistry parameters vary minimally both temporally and spatially. This relative uniformity is uncharacteristic of coastal habitats where natural fluctuations of pH, temperature, salinity and oxygen can occur on daily, tidal, seasonal and annual time scales [12–17]. Hence static seawater pH exposures do not accurately reflect the conditions that most mussel populations are likely to experience *in situ* either presently or under future OA scenarios. Recent field observations have shown this environmental variability can result in present-day situations in which seawater pH exceeds OA predictions for the end of the century [18,19], with pH deviations of up to one unit [9,16,17]. The magnitude of coastal fluctuations in seawater pH is predominately influenced by freshwater inputs (salinity), upwelling events, temperature, tidal cycles, photosynthesis and respiration [20,21]. These pH fluctuations are expected to intensify in the future, along with other global climatic changes [22], hence as OA progresses, seawater pH in coastal habitats may regularly drop to well below 7.6 by the year 2100 [23].

Unfortunately, our current understanding of how near-shore and intertidal marine species will respond to the natural variability of seawater pH in either present-day or near-future pH conditions, or how close these species are to their physiological limits, is very limited. Where fluctuating seawater pH/*p*CO_2_ conditions have been included in OA exposures, it has generally been found that responses are considerably different compared with those under static conditions. For example, Dufault *et al*. [24] found that recruits of the brooding reef coral, *Seriatopora caliendrum*, exposed to diurnally oscillating *p*CO_2_ grew 6–19% larger than those in static ambient or high *p*CO_2_ conditions. Frieder *et al*. [25] found the impact of low pH (pH 7.5) on early development of mussel larvae was reduced when pH fluctuated by 0.15 pH units around this low average pH value. Other studies, however, have found fluctuating conditions appear to enhance the impacts of OA [26,27]. While contrasting, these early results across a range of organisms all demonstrate that variable pH/*p*CO_2_ conditions can alter responses to OA conditions, highlighting the need to consider variability when conducting OA experiments for coastal species [28].

Here we test the hypothesis that a variable seawater pH regime will pose a greater physiological challenge compared with static conditions in the common mussel *M. edulis*. We first investigate the immediate acid–base and metabolic response of mussels to short-term changes in seawater pH to determine the rate of responses to external changes in seawater pH. We then investigate the consequences of a variable compared with a static pH regime on physiological responses over 2 weeks. We chose to use a semidiurnal pattern of fluctuations characteristic of tidally driven estuarine environments [13], where submerged mussels will experience a range of conditions. Indeed, Baumann *et al*. [16] show pH to be significantly correlated with tidal height in a temperate salt marsh, with ΔpH ≥ 1 unit on several occasions. Tidally driven variability in carbonate systems is likely to be important for many estuarine species and particularly for those comprising natural shellfish aquaculture globally, yet it has not been studied in detail to date. This approach is also relevant for submerged mussels living in tide pools, where the pH will vary primarily as a result of biological activity through each low-tide period when the pool is isolated, but will return to a nominal seawater pH condition when the tide is high [17].

## 2. Material and methods

### (a) Collection of animals

Adult *M. edulis* (55–73 mm shell length) were collected from a muddy estuary at Starcross, Devon, UK (50°37′03′ N 3°26′56′ W). Animals were first cleaned of barnacles then maintained in a recirculating system of artificial seawater (Tropic Marine, pH_NBS_ 8.10, salinity 33, at temperature 15 ± 0.5°C) with a photoperiod of 12 L : 12 D cycle. Mussels were held for a minimum of 7 d prior to experimentation and fed 5000 cells ml^−1^ of dried *Isochrysis* Instant Phyto (ZM Systems) daily.

### (b) Seawater manipulation

Artificial seawater was acidified to the desired pH level via the addition of CO_2_ gas using a computerized pH_NBS_ control system (Aqua Medic, Germany). Seawater pH (NBS scale) was additionally monitored with a Metrohm 826 pH_NBS_ mobile electrode and meter. Gentle aeration maintained oxygen levels close to 100% air-saturation. Seawater samples were collected at each sample time point (details below) for assessment of pH and dissolved inorganic carbon (DIC) (full methods in electronic supplementary material). The seawater carbonate chemistry data for the short-term changes in seawater pH are provided in the electronic supplementary material, tables S1 and S2, and for the medium-term exposure in table 1.

**Table 1.** Seawater carbonate chemistry from the 14-day experiment, showing mean ± s.d. for the stable treatments on day 0 and day 14. Temperature (temp.), salinity, pH and DIC were measured, while other carbonate parameters were calculated using CO_2_sys.

treatment	day	temp. (°C)	salinity	pH_NBS_	DIC (µmol kg^−1^)	TA (µmol kg^−1^)	*p*CO_2_ (µatm)	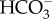 (µmol kg^−1^)	CO_3_^2−^(µmol kg^−1^)
pH 8.10 static	0	13.2 ± 0.1	31.7 ± 0.1	8.14 ± 0.01	2145 ± 9	2322 ± 10	439 ± 1	1992 ± 9	135 ± 1
pH 8.10 static	14	13.2 ± 0.1	30.1 ± 0.1	8.11 ± 0.00	1975 ± 34	2122 ± 36	441 ± 8	1845 ± 32	112 ± 2
pH 7.70 static	0	13.2 ± 0.0	31.6 ± 0.1	7.69 ± 0.01	2341 ± 92	2360 ± 90	1404 ± 79	2231 ± 88	53 ± 1
pH 7.70 static	14	13.2 ± 0.0	30.4 ± 0.1	7.69 ± 0.01	2081 ± 121	2100 ± 121	1276 ± 72	1985 ± 115	46 ± 3

### (c) Responses to short-term changes in seawater pH

Mussels were placed into tanks at a starting pH_NBS_ of 8.12 (*p*CO_2_ = 355 µatm, salinity 32.3, temperature 13.5 ± 0.5°C). The manipulation of gaseous CO_2_ gradually reduced seawater pH by one unit over a period of 6 h (to measure mussel response to acidification). CO_2_ manipulation was then stopped and the seawater pH was allowed to increase again by one unit over the following 6 h (to measure mussel recovery response). Seawater samples for DIC analysis and acid–base measurements of exposed mussels were taken every 30 min over each of the two 6 h periods (acidification and recovery). Six mussels were used for determination of acid–base response at each of the 26 sampling time points (i.e. 13 acidification and 13 recovery), with all mussels only being sampled once (to avoid any stress responses from the sampling process). Haemolymph (approx. 1 ml) from the posterior abductor muscle was extracted using a 21 g needle connected to a 2 ml syringe and immediately measured for pH_NBS_ (Metrohm 826 pH mobile electrode and meter). Haemolymph samples were then transferred to 100 µl glass micro-haematocrit capillary tubes sealed with paraffin oil and Hemato-Seal (Fisher) and placed on ice until subsequent analysis of total CO_2_ using a Corning 965 CO_2_ analyser (Corning Ltd., UK) calibrated with 10 mM NaHCO_3_. Acid–base parameters were calculated using a modified version of the Henderson–Hasselbalch equation using the first dissociation constant (p*K*) for carbonic acid and solubility constant (αCO_2_) for CO_2_ derived from Truchot [29] for invertebrate haemolymph.

Mussel metabolic responses to gradual reductions in seawater pH from pH_NBS_ 8.15 to 7.15 were measured during a separate exposure using mussels that had been starved for 3 days prior to the experiment (to avoid postprandial metabolism) and left to acclimate to the respirometry chambers overnight (to avoid handling stress). Mussels (*n* = 12) were placed in individual glass beakers (200 ml) initially containing seawater at pH_NBS_ 8.15. Oxygen consumption was measured for each mussel over 30 min before this seawater was replaced (flushed) with new seawater pre-gassed to the next experimental pH level (taking approx. 5 min for each water change). A total of nine measurements were taken over the gradual reduction before mussels were then immediately returned to ambient seawater (pH_NBS_ 8.20) to assess recovery of metabolic rate. Full details of the method are provided in the electronic supplementary material.

### (d) Physiological responses to fluctuating pH

To investigate the role of seawater pH variability in determining OA response, mussels (*n* = 16) were placed into one of four treatments: (1) static pH_NBS_ 8.1; (2) static pH_NBS_ 7.7; (3) fluctuating pH_NBS_ 8.1; and (4) fluctuating pH_NBS_ 7.7. An experimental pH_NBS_ value pf 7.7 was targeted to represent near-future OA under IPCC RCP 8.5 [22,30]. Each exposure system consisted of a 150 l header tank and 16 individual 1 l replicate tanks (i.e. one mussel per tank) in self-contained re-circulation. For the fluctuating treatments, dial compact 24 h timers were connected to the pH computers and set to replicate a semidiurnal tidal cycle (6 h on, 6 h off) to attain a fluctuating pH regime that first gradually reduced seawater over 6 h then allowed it to recover via aeration for the following 6 h (electronic supplementary material, table S3) (i.e. a 12 h cycle). Mussels were exposed to a total of 28 cycles of pH over the two-week exposure. Seawater was aerated and pH_NBS_, measured as previously described, was additionally recorded in each tank every 10 min for 24 h on a rotation, such that each tank was monitored for 24 h every 4 days (see electronic supplementary material, figure S1 and table S3). Mussels were fed as before, on days 0 and 7.

Oxygen consumption rates were measured after 14 days, as described above (and in electronic supplementary material). Briefly, each mussel was kept in its individual tank (to avoid handling stress) but the water supply was removed. After a 5 min settling period, *p*O_2_ was determined for a total of 2.5 h. Acid–base parameters were then measured as described above (after 14 days). The remaining haemolymph was immediately frozen in liquid nitrogen for further analysis (below). For the variable treatments, measurements were taken on the downwards phase at the mean pH value (either pH_NBS_ 8.10 or 7.70) to allow for direct comparisons with the static pH treatments.

### (e) Lysosomal stability and oxidative stress assays

The neutral red retention (NRR) assay was used as a measure of lysosomal stability of mussel haemocytes. This assay has been demonstrated to provide a useful indicator of general health status in *M. edulis*, correlating well with growth and reproduction [31]. Methodology followed that of Cajaraville *et al*. [32] (detailed in electronic supplementary material). Superoxide dismutase (SOD) is an important antioxidant enzyme catalysing the dismutation of superoxide anions and is often measured as an indicator of oxidative stress. SOD activity was quantified using the methodology of van der Oost *et al*. [33]. Lipid peroxidation was determined using the thiobarbituric acid reactive substances (TBARS) assay which quantifies malondialdehyde, a secondary product of lipid peroxidation, via its reaction with thiobarbituric acid following the methodology of Camejo *et al*. [34]. Full details of these assays are provided in the electronic supplementary material.

### (f) Statistical analysis

All data are presented as mean ± standard error (s.e.) and tested for normality using the Shapiro–Wilk Test. Normally distributed data for the 6 h exposure were analysed using a one-way ANOVA followed by the Holm–Sidak post hoc test. Where repeated measurements were taken from one mussel (6 h metabolic rate) a repeated-measures ANOVA was used. For data that failed the normality test (6 h haemolymph 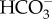 concentration) a Kruskal–Wallis test followed by Dunn's method post hoc test was used. A general linear model testing for effects of ‘pH’ (i.e. seawater pH_NBS_ of 8.1 or 7.7), ‘variability’ (static or fluctuating regime) and ‘pH × variability’ (to test for an interaction term) was used for the 14-day exposure endpoints. Data were analysed using Sigma Plot v. 12 and SPSS v. 24.

## 3. Results

### (a) Short-term responses to changes in seawater pH

**Figure 1.**
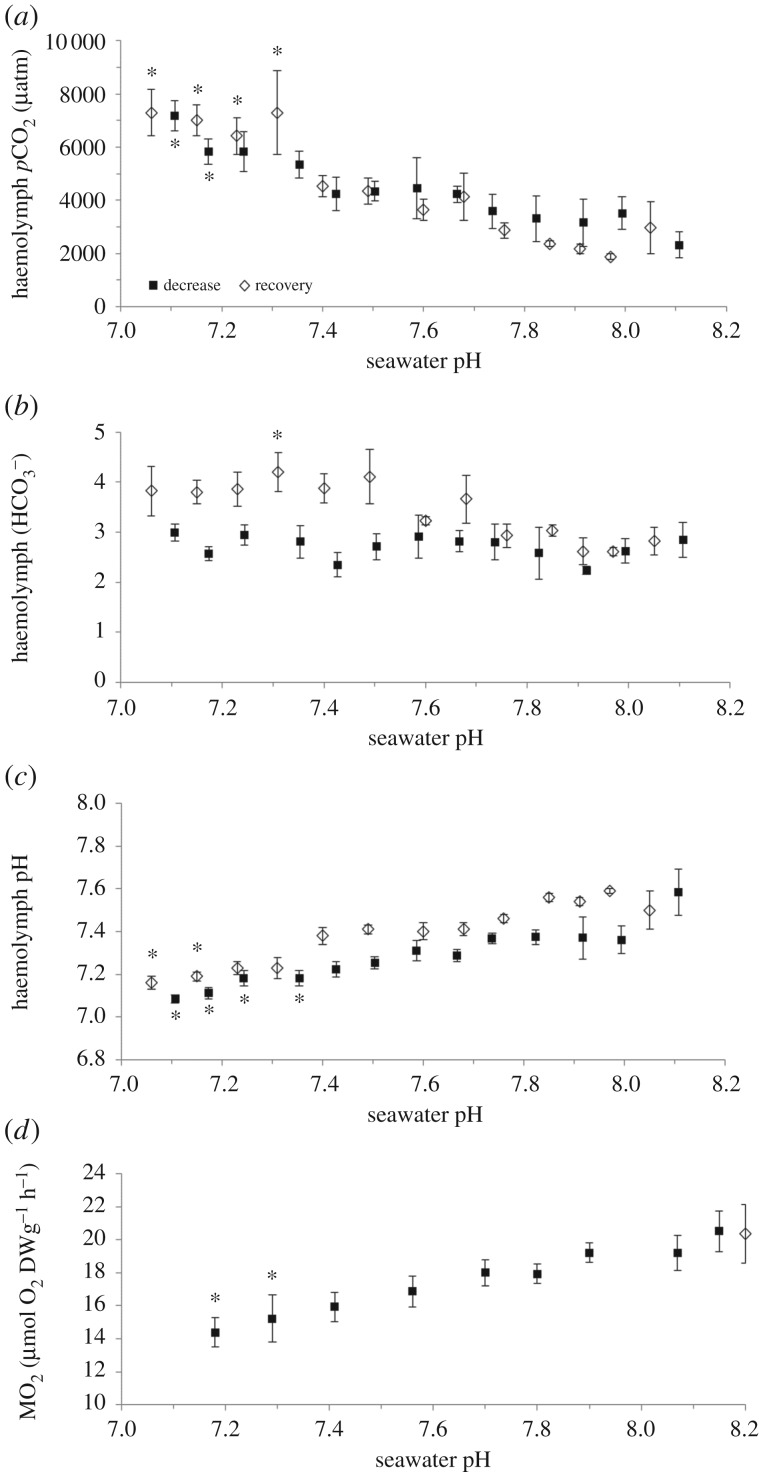
Acid–base parameters in the haemolymph (*a–c*) and metabolic rate (*d*) of *M. edulis* over a 6 h gradual exposure to decreasing seawater pH (dark grey square symbols) and recovery (light grey diamond symbols). Data shown as mean ± s.e. Asterisk represents a significant difference from that measured at seawater pH 8.11.

Acidification from pH_NBS_ 8.11 to 7.11 resulted in a threefold increase in haemolymph *p*CO_2_ (figure 1*a*; *F* = 3.894, *p* < 0.001). No accompanying change in haemolymph bicarbonate levels was observed (figure 1*b*; *F* = 0.588, *p* = 0.844), leading to a significant decrease in haemolymph pH from pH_NBS_ 7.58 to 7.09 (figure 1*c*; *F* = 0.699, *p* < 0.001). Mussels steadily recovered from this acidosis during the following 6 h increase in seawater pH, with haemolymph *p*CO_2_ significantly decreasing (figure 1*a*; *F* = 8.207, *p* = < 0.001) and pH increasing back to levels similar to those measured at ambient seawater pH (pH_NBS_ 8.10) (figure 1*c*; *F* = 14.679, *p* < 0.001). Haemolymph bicarbonate (figure 1*b*) started the 6 h ‘recovery’ phase significantly higher (*F* = 3.054, *p* = 0.02) but reached values comparable to those in ambient pH animals halfway through the recovery of seawater pH. Metabolic rate was also significantly affected by acidification (figure 1*d*; *F* = 3.21, *p* < 0.001), decreasing by 30% over a one-unit decrease in seawater pH (6 h) and recovering immediately when exposed to ambient seawater (approx. 15 min).

### (b) Physiological responses to fluctuating pH

The pH fluctuating regimes are shown in electronic supplementary material, figure S1. We were unable to raise the pH of the control in the fluctuating conditions greater than to about 8.2 solely by aeration, which resulted in an uneven fluctuation above and below the average value of 8.1, as we had aimed to achieve. To further increase the pH in this system would require carbonate chemistry to be manipulated in ways unrepresentative of CO_2_-induced changes. Details of the fluctuating regimes are therefore as follows: pH_NBS_ 8.1 fluctuating had a mean ± s.d. of 7.92 ± 0.20, max = 8.23, min = 7.66; pH_NBS_ 7.7 fluctuating had a mean ± s.d. of 7.70 ± 0.32, max = 8.13, min = 7.26 (electronic supplementary material, table S3).

**Figure 2.**
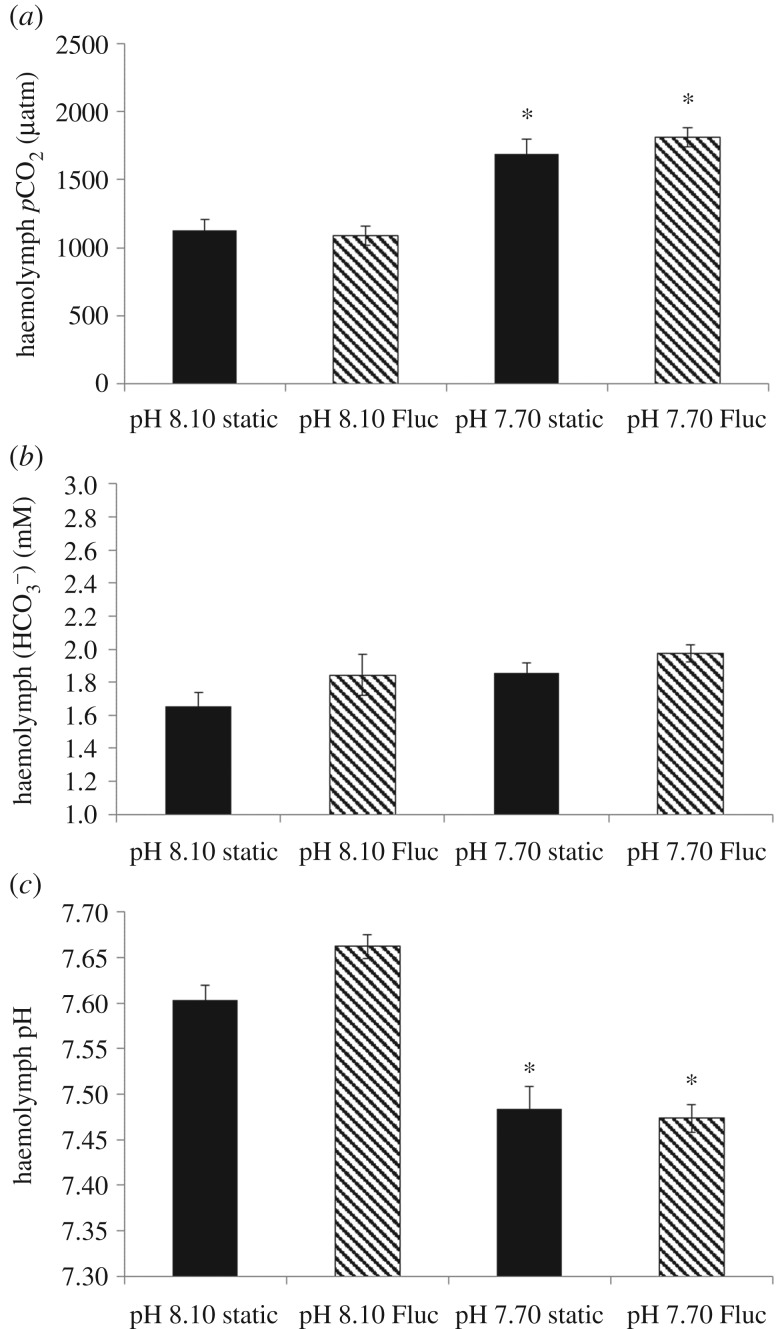
Acid–base parameters in the haemolymph of *M. edulis* following a 14-day exposure to control and lowered pH in static and fluctuating (Fluc) pH regimes: (*a*) haemolymph *p*CO_2_; (*b*) haemolymph bicarbonate concentration (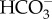); and (*c*) haemolymph pH. Data shown as mean ± s.e. Asterisk represents significant differences from the static pH 8.10 treatment.

Similar to the response observed during the short-term experiment, haemolymph *p*CO_2_ levels were significantly affected by seawater pH, and were 43% and 48% higher in the pH_NBS_ 7.70 static and fluctuating treatments, respectively, compared with the pH_NBS_ 8.10 static and fluctuating treatments (figure 2*a*; GLM for ‘pH’; *F* = 64.487, *p* < 0.001). A fluctuating regime had no effect on haemolymph *p*CO_2_ (GLM for ‘variability’: *F* = 0.241, *p* = 0.625; for ‘pH × variability’: *F* = 1.076, *p* = 0.304). There was a small but significant increase in haemolymph bicarbonate levels driven by seawater pH (figure 2*b*; GLM for ‘pH’: *F* = 4.032, *p* = 0.049), with no effect of a fluctuating regime (GLM for ‘variability’: *F* = 3.374, *p* = 0.71; for ‘pH × variability’: *F* = 0.178, *p* = 0.675). Haemolymph pH was significantly decreased by seawater pH (figure 2*c*; GLM for ‘pH’: *F* = 70.882, *p* < 0.001), where pH decreased by up to 0.19 units. There was no effect of a fluctuating regime on haemolymph pH (GLM for ‘variability’: *F* = 1.737, *p* = 0.193; for ‘pH × variability’ interaction: *F* = 3.56, *p* = 0.064).

**Figure 3.**
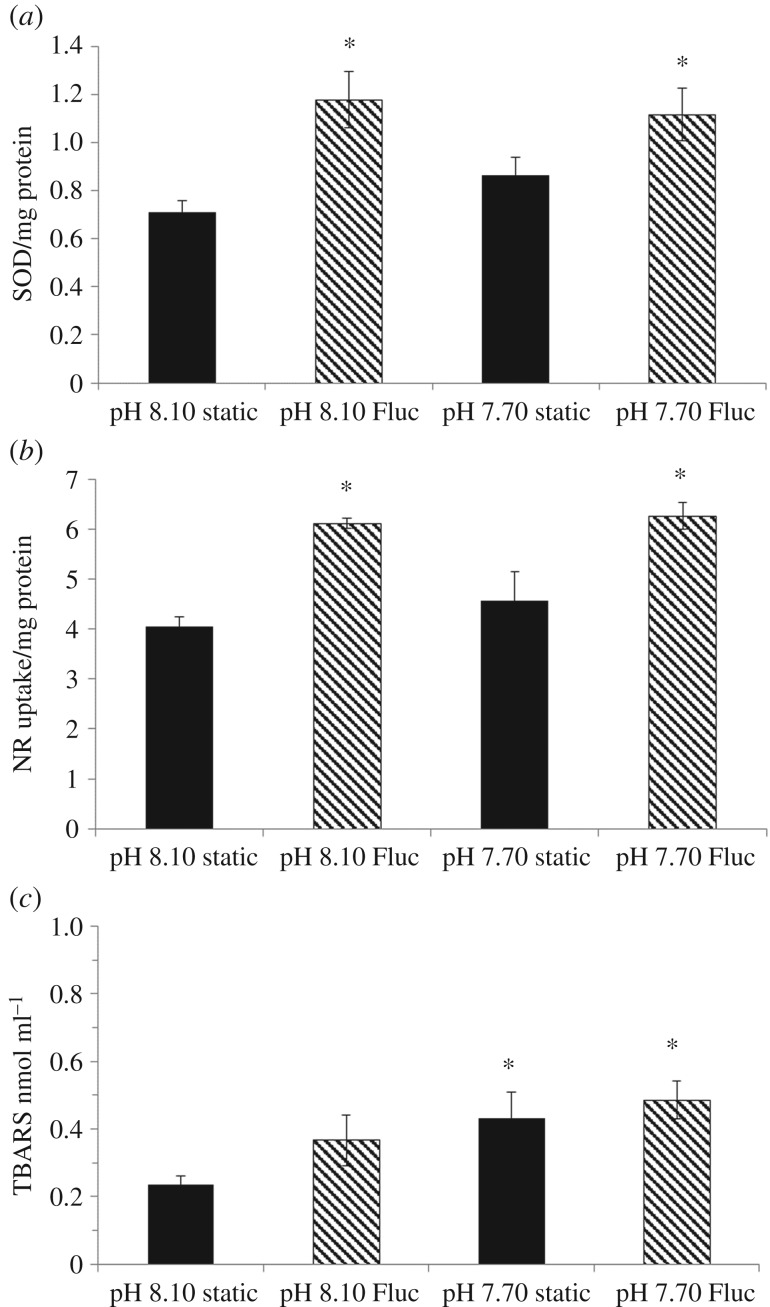
Health indicators measured in the haemolymph of *M. edulis* following a 14-day exposure to control and lowered pH conditions in static and fluctuating (Fluc) pH regimes: (*a*) activity of the antioxidant enzyme superoxide dismutase (SOD); (*b*) cell viability measured as neutral red retention; and (*c*) levels of thiobarbituric acid reactive substances (TBARS). Data shown as mean ± s.e. Asterisk represents significant differences from the static pH_NBS_ 8.10 treatment.

**Figure 4.**
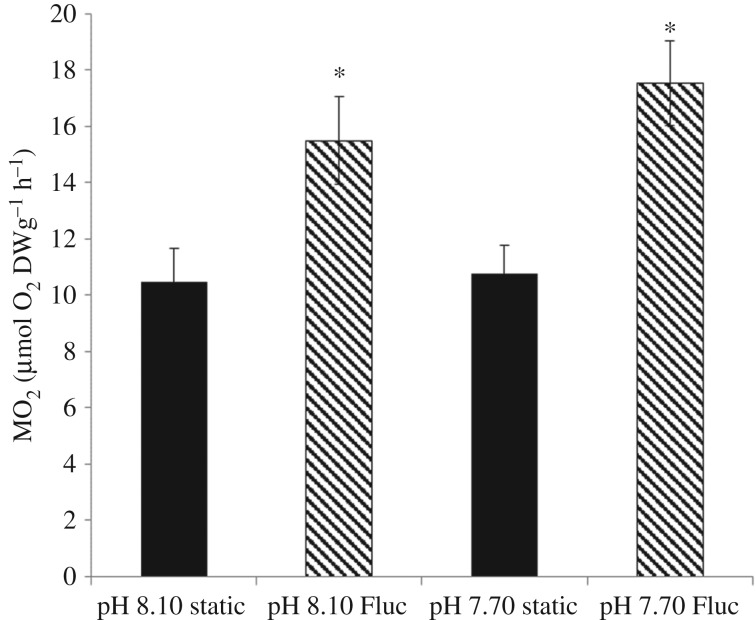
The metabolic rate of *M. edulis* following a 14-day exposure to control and lowered pH conditions in static and fluctuating (Fluc) pH regimes (data as mean ± s.e.). Asterisk represents significant differences from the static pH_NBS_ 8.10 treatment.

Haemolymph SOD activity was significantly increased by a fluctuating pH/*p*CO_2_ regime (figure 3*a*; GLM for ‘variability’: *F* = 14.11, *p* < 0.001), showing an increase of 49% and 44% in the fluctuating pH_NBS_ 8.10 and pH_NBS_ 7.70 treatments, respectively, compared with the pH_NBS_ 8.10 static treatment. There was no effect of pH treatment on SOD activity. Similarly, NRR was significantly influenced by a fluctuating regime (GLM for ‘variability’: *F* = 26.66, *p* < 0.001) and was higher by 41% and 43% in the pH_NBS_ 8.10 and 7.70 fluctuating treatments, respectively, compared with pH_NBS_ 8.10 static treatment (figure 3*b*). NRR was not significantly influenced by pH treatment (GLM for ‘pH’: *F* = 0.628, *p* = 0.431; no interaction ‘pH × variability’: *F* = 0.683, *p* = 0.412). TBARS was significantly elevated by pH treatment (figure 3*c*; GLM for ‘pH’: *F* = 6.916, *p* = 0.011), with no significant influence of a fluctuating regime on lipid peroxidation and no interaction term (GLM for ‘variability’: *F* = 2.350, *p* = 0.131; for ‘pH × variability’: *F* = 0.429, *p* = 0.515). In contrast to the shorter (6 h), experiment, there was no significant effect of pH treatment on mussel MO_2_ after 2 weeks (figure 4; GLM for ‘pH’; *F* = 0.76, *p* = 0.388). However, there was a significant effect of a fluctuating pH regime, with increased MO_2_ of 39% and 50% in the fluctuating pH_NBS_ 8.10 and pH_NBS_ 7.70 treatments, respectively, compared with pH_NBS_ 8.10 static (figure 4; GLM for ‘variability’: *F* = 19.68, *p* < 0.001; for ‘pH × variability’ interaction: *F* = 0.444, *p* = 0.509).

## 4. Discussion

Our data clearly show that exposure to fluctuating seawater carbonate chemistry elicits a very different set of physiological responses than exposures to static conditions for both ambient and lowered pH exposures in *Mytilus edulis,* collected from an estuarine habitat. Importantly, the variable treatments elicited greater oxidative stress and a greater effect on metabolic rate than the static seawater conditions for both ambient and near-future *p*CO_2_ levels, suggesting variable environments are more energetically costly for individuals than static ones.

Consistent with other physiological studies, our results show that *M. edulis* have a limited ability to acid–base regulate during both short- (6 h) and medium-term (14 day) changes in seawater pH. An increase in seawater *p*CO_2_ caused a direct and immediate increase in mussel haemolymph *p*CO_2_, with levels reaching up to 1816 µatm (at the mid-point of the pH cycle) in the 2-week exposure and up to 7200 µatm at the highest seawater *p*CO_2_ value (pH_NBS_ 7.06) in the 6 h experiment. An increase in extracellular *p*CO_2_ under OA conditions has been observed in a number of marine organisms [35,36], including *M. edulis*, where extracellular *p*CO_2_ increased linearly with seawater *p*CO_2_ [8]. Mussel haemolymph *p*CO_2_ values as high as 5724 µatm have previously been recorded in *M. galloprovincialis* exposed to seawater pH of 7.3 [37]. A corresponding reduction in haemolymph pH was observed resulting from the elevated *p*CO_2_ levels, which was then immediately reversed when seawater conditions were gradually returned to their starting conditions. These observations are in agreement with previous studies reporting that *M. edulis* cannot actively avoid acid–base disturbances [8–10,38], but conforms to changes in *p*CO_2_ of the external medium. The short-term extracellular accumulation of bicarbonate ions is often reported as an efficient mechanism in stabilizing extracellular pH in active marine ectotherms [39]. Although we saw a trend of increasing haemolymph 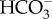 concentrations after 2 weeks of acclimation, this was not enough to compensate extracellular pH. The inability to compensate extracellular acid–base disturbances potentially has negative impacts on growth and calcification under extended exposures to OA.

In addition to influencing the acid–base status of mussels over short-term exposures, increasing seawater *p*CO_2_ also led to a reduction in metabolic rate, with an immediate recovery of oxygen consumption when returned to ambient seawater conditions (pH_NBS_ 8.20). This could be driven by a number of mechanisms, such as metabolic suppression or a reduction in enzyme efficiency under the acidic conditions. Similar responses have been recorded in a number of invertebrates; for example, a reduction in metabolic rate in response to reduced extracellular pH in *M. galloprovincialis* was suggested to result from an inhibition of net proton transport across the cell membrane [37,40]. Metabolism may also be influenced by reductions in intracellular pH by inhibiting enzymatic activity [41].

Interestingly, the metabolic response of *M. edulis* to OA treatments over the 2-week exposure differed considerably when compared with their short-term responses. We found no significant effect of OA on mussel metabolic rate after 2 weeks of static exposure. However, there was a significant increase in metabolic rate by 39% and 50% under both fluctuating regimes of pH_NBS_ 8.10 and 7.70, respectively, compared with those exposed to static pH_NBS_ 8.10. Hence, the metabolic suppression in response to short-term changes in seawater pH was replaced with no overall effect of static OA but a clear energetic cost of fluctuating pH conditions over the longer exposure. Other studies have shown food availability can influence individuals' OA response because of these changes in metabolic cost [9] and that increased food, when available, can provide the additional energy required. Our findings align with recent work on the influence of tidal biological rhythms on metabolic rates of the ghost shrimp, *Neotrypaea uncinata*, where metabolic rates were higher under simulated fluctuating tidal conditions compared with static conditions in the laboratory [42]. The difference in metabolic rate between static and fluctuating OA conditions may partly be explained via changes in energy demand associated with intracellular acid–base regulation. As shown in *M. galloprovincialis*, intracellular pH can take a few days to be fully compensated [37], which could explain the difference between the immediate reduction in metabolic rate compared with increases in metabolic rate over the longer exposure.

Elevated stress responses under the fluctuating seawater pH regimes were also observed for a number of the stress-related biomarkers within the haemolymph. While static OA conditions had no effect on the activity levels of the antioxidant enzyme superoxide dismutase (SOD), both ambient and OA fluctuating conditions induced a significant increase in SOD activity, of 49% and 44%, respectively. This is in agreement with previous studies where no change in SOD activity was observed in *M. edulis* and *M. galloprovincialis* exposed to static OA conditions [43,44]. Despite the increase in SOD activity measured in mussels from both of the fluctuating treatments, an increase in lipid peroxidation was seen in the OA static and both fluctuating treatments. This strongly suggests that fluctuating pH seawater conditions exert a much greater oxidative stress on individuals than static conditions, both triggering an increase in SOD activity and overwhelming this response to accumulate lipid peroxidation. Our data suggest that the static OA conditions did not exert enough oxidative stress to trigger an antioxidant response in the mussels, resulting in low levels of lipid peroxidation accumulating over the 14 days.

We also found that while static OA conditions had no significant effect on NRR, a significant increase in NRR was measured in response to both fluctuating treatments. This contrasts with the previous work of Beesley *et al*. [45], who found *M. edulis* exposed to static seawater pH_NBS_ treatments of 7.80, 7.60 and 6.80 showed a significant reduction in NRR; however, this study used a much longer exposure time of 60 days, which may account for this difference. The increased NRR in fluctuating treatments may again be explained by the upregulation of SOD production translating into an intensification of the immune system which decreases the permeability of the lysosome membrane. However, this does appear to come at an energetic cost as shown via the significant increase in metabolic rate under both fluctuating regimes.

Running experiments using a fluctuating system posed two significant challenges: First, in order to create replicate fluctuating systems we used just one header tank per treatment to create the conditions before the manipulated water was then fed into individual mussel experimental tanks. While we acknowledge the limitation of this design, we believe that by careful continual monitoring of all the tanks, we did not see any tank effects and all results presented here are in response to the various pH treatments. The second challenge was that the two fluctuating conditions were achieved by bubbling with CO_2_ to reduce the pH and aerating the tanks to allow them to recover back up to higher levels. This method was successful in the pH 7.7 treatment, because the mean pH was already lower than ambient. However, it was difficult to raise the pH above the ambient 8.1 just using bubbling. Thus the 8.1 fluctuating treatment actually had a smaller overall pH range compared with the 7.1 fluctuating treatment. Nevertheless, we still achieved fluctuating regimes that were significantly different from the stable regimes, and therefore have confidence in our results.

Overall, our data clearly show significantly different physiological responses of organisms exposed to a fluctuating compared with a static seawater pH regime. Analysis of several health parameters suggests exposure to a fluctuating pH regime is more energetically demanding for coastal organisms than static pH conditions. The few studies to date using a fluctuating pH cycle all found that the responses of their test organisms differed between static and fluctuating conditions. In some cases fluctuating conditions appeared to alleviate the effects of elevated *p*CO_2_ [25] while others found that fluctuating conditions exacerbated a negative OA effect [26,27]. With seawater pH fluctuations expected to increase in intensity and frequency over the coming decades [22], it is vital to understand how coastal organisms respond to current variability in order to predict their responses to additional OA. Our results highlight the difficulties in extrapolating outcomes of static pH exposures for species which experience large fluctuations in their physiochemical environment, with static pH experiments potentially incorrectly estimating coastal species responses to future OA. The precise nature of the fluctuations experienced currently by natural populations remain poorly understood, but are likely to vary considerably with habitat, location and time of year. Collecting good-quality data on the conditions currently experienced by intertidal and subtidal populations is urgent to better understand the range and periodicity of these fluctuations and enhance future experimental designs if we are to suitably predict coastal organism responses to future OA.

## Supplementary Material

Supporting information

## Supplementary Material

Tables3_24hourpHmonitoring.csv

## Data accessibility

The datasets supporting this article have been uploaded as part of the electronic supplementary material.
