# Supplementary material for: Fluctuating seawater pH/pCO2 regimes are more energetically expensive than static pH/pCO2 levels in the mussel Mytilus edulis
Source: Proc Biol Sci. 2017 Oct 18;284(1865):20171642. doi: 10.1098/rspb.2017.1642 (PMC5666100; doi:10.1098/rspb.2017.1642)
Supplement: Supporting information [file rspb20171642supp1.pdf]

Supporting Information: Fluctuating seawater pH/ pCO$_2$ regimes are more energetically expensive than constant pH/ pCO$_2$ levels in the mussel *Mytilus edulis*

Supporting Methods:

Seawater carbonate chemistry analysis

Samples of 12 mL were preserved with saturated $HgCl_2$ and analysed for DIC using a custom built system based on Friederich et al. [1], following methodology from Lewis et al. [2] and using Dickson reference material as standards. DIC data outputs were analysed using Logger Pro version 3.2 software with a measurement precision of ± 3 µM. Total alkalinity and pCO_2 were calculated using CO2sys [3] using temperature, salinity, pH_{NBS} and DIC data with K1 and K2 values refitted by Dickson and Millero [4] from Mehrbach [5], and KSO_4 determined by Dickson [6].

Mussel metabolic rate measurement for the short term exposures

The partial pressure of oxygen (pO_2) was assessed using a fibre-optic oxygen sensor (FireStingO2) and recorded by the Pyro-Science Fire Sting logger (v2.363 2012). Oxygen concentration remained above 80% saturation during each measurement and mussels were given 5 minutes after each water change to settle before the next set of measurements. Three blanks were also included to assess any potential for bacterial respiration or diffusive gain of oxygen from the atmosphere. Immediately after the final sampling point mussels were removed and seawater was weighed (Scout Pro 600g OHAUS) to accurately record the incubation volume. Shell length was recorded for each mussel before being placed into a drying oven for 48 h at 60 °C to determine shell and organic dry weight. Metabolic rate was calculated using organic dry weights for each mussel.

Neutral red retention assay

Haemolymph was incubated for 45 minutes at room temperature in a sealed poly-L-lysine treated microtiter plate. Adhered haemocytes were incubated with 0.2% NR solution in phosphate buffered saline (PBS) for 3 h before excess NR was discarded. Cells were washed with PBS before the addition of acidified ethanol (1% acetic acid, 50% ethanol). The absorbance of NR was then measured spectrophotometrically at 540 nm using a microplate reader (Tecan NanoQuant infinite M200PRO).

Oxidative stress assays

To measure superoxide dismutase (SOD) activity in mussel haemolymph; a Na_2CO_3/$NaHCO_3$ buffer (pH_{NBS} 10.2) and NBT solution (0.1 mM Xanthine, 0.1 mM EDTA, 0.05 mg BSA, 0.025 mM NBT) were added together with haemolymph to a microtiter plate. Xanthine oxidase in a Na_2CO_3/$NaHCO_3$ buffer solution was then added to all wells before the absorbance was measured spectrophotometrically at 573 nm for 25 cycles and compared to a standard curve (0-3 U mL^{-1} SOD). Lipid peroxidation was determined using the thiobarbituric acid reactive substances (TBARS) assay which quantifies malondialdehyde (MDA), a secondary product of lipid peroxidation, via its reaction with thiobarbituric acid following the methodology of Camejo et al. [7]. Standards were made using serial dilutions of 1, 1, 3, 3, tetraethoxypropane and ethanol. Both the SOD and TBARS assays were normalised to protein content using Bradford protocol and BSA as standards [8].

Table S1: Seawater carbonate chemistry for the short-term acid-base responses of *Mytilus edulis* to a reduction in seawater pH followed by pH recovery. Temperature (Temp.), salinity, pH and DIC were measured, while other carbonate parameters were calculated using CO2sys. All data is shown as mean ± SD.

Time (h)	Temp. (°C)	Salinity	pH_{NBS}	DIC (µmol kg^{-1})	TA (µmol kg^{-1})	pCO_2 (µatm)	HCO_3^- (µmol kg^{-1})	CO_3^{2-} (µmol kg^{-1})
0.0	13.4 ± 0.2	32.3 ± 0.0	8.12 ± 0.0	2239 ± 17	2483 ± 18	354 ± 3	2043 ± 15	182 ± 1
0.5	13.5 ± 0.5	32.3 ± 0.1	8.06 ± 0.01	2355 ± 81	2574 ± 87	431 ± 15	2170 ± 75	168 ± 6
1.0	13.5 ± 0.1	32.3 ± 0.1	7.97 ± 0.01	2368 ± 85	2547 ± 89	526 ± 19	2206 ± 79	141 ± 5
1.5	13.5 ± 0.1	32.2 ± 0.1	7.90 ± 0.01	2374 ± 50	2515 ± 57	648 ± 27	2231 ± 57	118 ± 0
2.0	13.5 ± 0.2	32.3 ± 0.1	7.81 ± 0.00	2414 ± 33	2524 ± 34	807 ± 11	2283 ± 31	100 ± 1
2.5	13.5 ± 0.1	32.3 ± 0.0	7.72 ± 0.00	2746 ± 370	2520 ± 59	1009 ± 24	2321 ± 55	82 ± 2
3.0	13.5 ± 0.0	32.3 ± 0.1	7.64 ± 0.01	2469 ± 75	2518 ± 76	1228 ± 37	2351 ± 71	69 ± 2
3.5	13.5 ± 0.1	32.2 ± 0.1	7.55 ± 0.01	2340 ± 59	2358 ± 61	1450 ± 13	2230 ±56	53 ± 3
4.0	13.4 ± 0.1	32.3 ± 0.1	7.45 ± 0.00	2477 ± 117	2465 ± 115	1907 ± 90	2357 ± 111	45 ± 2
4.5	13.5 ± 0.1	32.3 ± 0.0	7.38 ± 0.01	2493 ± 85	2458 ± 84	2249 ± 77	2365 ± 81	38 ± 1
5.0	13.5 ± 0.1	32.3 ± 0.2	7.24 ± 0.02	2551 ± 87	2467 ± 83	3148 ± 107	2398 ± 81	28 ± 1
5.5	13.4 ± 0.1	32.3 ± 0.1	7.17 ± 0.01	2549 ± 144	2438 ± 137	3670 ± 207	2380 ± 134	24 ± 1
6.0	13.5 ± 0.0	32.3 ± 0.0	7.12 ± 0.01	2582 ± 152	2448 ± 143	4146 ± 244	2396 ± 141	21 ± 1
6.5	13.5 ± 0.1	32.1 ± 0.1	7.06 ± 0.01	2337 ± 55	2191 ± 58	4275 ± 31	2150 ± 56	17 ± 1
7.0	13.5 ± 0.0	32.1 ± 0.1	7.15 ± 0.02	2340 ± 97	2228 ± 100	3559 ± 20	2178 ± 96	20 ± 2
7.5	13.5 ± 0.2	32.1 ± 0.1	7.22 ± 0.03	2255 ± 35	2174 ± 44	2913 ± 137	2116 ± 39	24 ± 2
8.0	13.5 ± 0.0	32.1 ± 0.1	7.29 ± 0.02	2280 ± 16	2220 ± 22	2548 ± 105	2151 ± 18	28 ± 2
8.5	13.5 ± 0.1	32.1 ± 0.1	7.37 ± 0.03	2288 ± 53	2254 ± 61	2113 ± 87	2169 ± 54	34 ± 3
9.0	13.5 ± 0.0	32.1 ± 0.1	7.46 ± 0.01	2247 ± 54	2239 ± 55	1712 ± 12	2138 ± 51	41 ± 2
9.5	13.5 ± 0.2	32.1 ± 0.1	7.56 ± 0.01	2249 ± 62	2271 ± 66	1347 ± 7	2143 ± 59	52 ± 3
10.0	13.5 ± 0.0	32.1 ± 0.1	7.66 ± 0.01	2197 ± 68	2248 ± 71	1057 ± 15	2092 ± 64	63 ± 3
10.5	13.5 ± 0.1	32.1 ± 0.1	7.76 ± 0.03	2125 ± 49	2208 ± 41	801 ± 72	2015 ± 50	78 ± 3
11.0	13.5 ± 0.0	32.1 ± 0.1	7.85 ± 0.02	2126 ± 4	2238 ± 4	655 ± 35	2006 ± 7	94 ± 5
11.5	13.5 ± 0.1	32.1 ± 0.1	7.91 ± 0.02	2104 ± 40	2238 ± 27	562 ± 59	2975 ± 44	107 ± 7
12.0	13.5 ± 0.0	32.1 ± 0.1	7.97 ± 0.03	2076 ± 38	2235 ± 28	474 ± 42	1936 ± 43	121 ± 6
12.5	13.5 ± 0.1	32.1 ± 0.1	8.05 ± 0.01	2061 ± 38	2255 ± 33	387 ± 21	1902 ± 40	143 ± 2

Table S2: Seawater carbonate chemistry for the experiment on short-term metabolic response in *Mytilus edulis* to a reduction in seawater pH followed by a recovery in pH. Temperature (Temp.), salinity, pH and DIC were measured, while other carbonate parameters were calculated using CO2sys. All data is shown as mean ± SD.

Time (h)	Temp. (°C)	Salinity	pH_{NBS}	DIC (µmol kg^{1})	TA (µmol kg^{-1})	*p*CO□ (µatm)	HCO□⁻ (µmol kg^{1})	CO□²⁻ (µmol kg^{-1})
0.00	12.9 ± 0.0	31.6 ± 0.0	8.15 ± 0.00	2103 ± 26	2343 ± 29	309 ± 4	1915 ± 24	175 ± 2
0.75	12.9 ± 0.0	31.6 ± 0.0	8.06 ± 0.01	2267 ± 25	2469 ± 24	421 ± 12	2096 ± 26	154 ± 1
1.50	12.9 ± 0.0	31.6 ± 0.0	7.90 ± 0.01	2263 ± 28	2393 ± 25	618 ± 18	2129 ± 27	108 ± 0
2.25	12.9 ± 0.0	31.6 ± 0.0	7.80 ± 0.00	2319 ± 27	2415 ± 27	793 ± 9	2197 ± 25	90 ± 1
3.00	12.9 ± 0.0	31.6 ± 0.0	7.71 ± 0.01	2350 ± 55	2415 ± 58	1005 ± 7	2236 ± 52	73 ± 3
3.75	12.9 ± 0.0	31.6 ± 0.0	7.56 ± 0.00	2431 ± 56	2449 ± 56	1454 ± 33	2317 ± 53	54 ± 1
4.50	12.9 ± 0.0	31.6 ± 0.0	7.40 ± 0.00	2448 ± 54	2416 ± 53	2108 ± 47	2324 ± 52	38 ± 1
5.25	12.9 ± 0.0	31.6 ± 0.0	7.29 ± 0.01	2414 ± 53	234 ± 48	2691 ± 101	2276 ± 48	28 ± 0
6.00	12.9 ± 0.0	31.6 ± 0.0	7.16 ± 0.00	2485 ± 43	2368 ± 41	3649 ± 64	2315 ± 41	22 ± 0
6.25	12.9 ± 0.0	31.6 ± 0.0	8.20 ± 0.00	2244 ± 199	2525 ± 217	291 ± 26	2024 ± 180	208 ± 181

Table S3: Seawater pH_{NBS} measured every 10 minutes over 24 hour periods on alternative days in each of the treatments during the two week experiment investigating the response in *Mytilus edulis* to fluctuating versus static pH conditions.

File: Tables3_24hourpHmonitoring.xls

Figure S1: Mean seawater pH_{NBS} conditions over a 24 hour period in the treatments in (a) static and (b fluctuating conditions. Errors bars represent standard deviation.

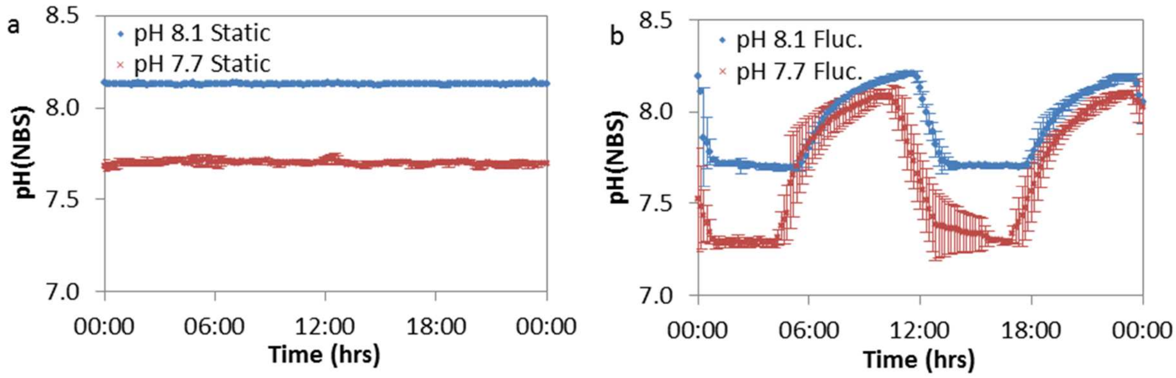

Supporting References:

1. Friederich G.E., Walz P.M., Burczynski M.G., Chavez F.P. 2002 Inorganic carbon in the central California upwelling system during the 1997-1999 El Nino-La Nina event. *Progress in Oceanography* **54**(1-4), 185-203. (doi:Pii s0079-6611(02)00049-610.1016/s0079-6611(02)00049-6).

2. Lewis C., Clemow K., Holt W.V. 2013 Metal contamination increases the sensitivity of larvae but not gametes to ocean acidification in the polychaete *Pomatoceros lamarckii* (Quatrefages). *Marine Biology* **160**(8), 2089-2101.

3. Pierrot D., Lewis E., Wallace D.W.R. 2006 MS Excel program developed for CO2 system calculations.

4. Dickson A.G., Millero F.J. 1987 A comparison of the equilibrium-constants for the dissociation of carbonic-acid in seawater media. *Deep-Sea Research Part a-Oceanographic Research Papers* **34**(10), 1733-1743. (doi:10.1016/0198-0149(87)90021-5).

5. Mehrbach C., Culberso.Ch, Hawley J.E., Pytkowic.Rm. 1973 Measurement of apparent dissociation-constants of carbonic-acid in seawater at atmospheric-pressure. *Limnology and Oceanography* **18**(6), 897-907.
6. Dickson A.G. 1990 Standard potential of the reaction - $AgCl(s)+1/2H_2(g)=Ag(s)+HCl(aq)$ and the standard acidity constant of the ion HSO_4^- in synthetic sea-water from 273.15-k to 318.15-k. *Journal of Chemical Thermodynamics* **22**(2), 113-127. (doi:10.1016/0021-9614(90)90074-z).
7. Camejo G., Wallin B., Enojärvi M. 1999 Analysis of oxidation and 610 antioxidants using microtiter plate. In *Free radical and antioxidant protocols Methods in molecular biology* (ed. D A.), pp. 377–387. Totowa, NJ,, Humana, .
8. Bradford M.M. 1976 Rapid and sensitive method for quantitation of microgram quantities of protein utilizing principle of protein-dye binding. *Analytical Biochemistry* **72**(1-2), 248-254. (doi:10.1006/abio.1976.9999).
